# Understanding the persistence of vertical (stand-alone) HIV clinics in the health system in Uganda: a qualitative synthesis of patient and provider perspectives

**DOI:** 10.1186/s12913-018-3500-4

**Published:** 2018-09-05

**Authors:** Henry Zakumumpa, Joseph Rujumba, Japheth Kwiringira, Jepchirchir Kiplagat, Edith Namulema, Alex Muganzi

**Affiliations:** 1 School of Public Health, Makerere University, Kampala, Uganda; 2 School of Medicine, Makerere University, Kampala, Uganda; 3 Department of Sociology, Kyambogo University, Kampala, Uganda; 4 College of Health Sciences, Moi University, Eldoret, Kenya; 5 Home care and counselling department, Mengo Hospital, Kampala, Uganda; 6 The Infectious Diseases Institute, Makerere University, Kampala, Uganda

**Keywords:** Health systems, Service delivery, HIV, Implementation research, Antiretroviral therapy, Integration, Global health initiatives

## Abstract

**Background:**

Although there is mounting evidence and policy guidance urging the integration of HIV services into general health systems in countries with a high HIV burden, vertical (stand-alone) HIV clinics are still common in Uganda. We sought to describe the specific contexts underpinning the endurance of vertical HIV clinics in Uganda.

**Methods:**

A qualitative research design was adopted. Semi-structured interviews were conducted with the heads of HIV clinics, clinicians and facility in-charges (*n = 78*), coupled with eight focus group discussions (64 participants) with patients from 16 health facilities purposively selected, from a nationally-representative sample of 195 health facilities across Uganda, because they run stand-alone HIV clinics. Data were analyzed by thematic approach as guided by the theory proposed by Shediac-Rizkallah & Bone (1998) which identifies; *Intervention characteristics, organizational context, and broader environment factors* as potentially influential on health programme sustainability.

**Results:**

*Intervention characteristics:* Provider stigma was reported to have been widespread in the integrated care experience of participating health facilities which necessitated the establishment of stand-alone HIV clinics. HIV disease management was described as highly specialized which necessitated a dedicated workforce and vertical HIV infrastructure such as counselling rooms. *Organizational context:* Participating health facilities reported health-system capacity constraints in implementing integrated systems of care due to a shortage of ART-proficient personnel and physical space, a lack of laboratory capacity to concurrently conduct HIV and non-HIV tests and increased workloads associated with implementing integrated care. *Broader environment factors:* Escalating HIV client loads and external HIV funding architectures were perceived to have perpetuated verticalized HIV programming over the past decade.

**Conclusion:**

Our study offers in-depth, contextualized insights into the factors contributing to the endurance of vertical HIV clinics in Uganda. Our analysis suggests that there is a complex interaction in supply-side constraints (shortage of ART-proficient personnel, increased workloads, laboratory capacity deficiencies) and demand-side factors (escalating demand for HIV services, psychosocial barriers to HIV care) as well as the specialized nature of HIV disease management which pose challenges to the integrated-health services agenda.

## Background

Although there is no consensus on the definition of the term health service integration [1, 2], the WHO describes service integration as ‘delivery of services or multiple interventions together on the same patient visit by the same health worker or clinical team’ [3]. In the context of HIV services, Odeny et al., [4] define integration as ‘co-location and sharing of services and resources for HIV care and primary care, such as clinic space, clinicians, health education, pharmacy, laboratory services, and training’.

The integration of HIV services into the general health system in countries with a high HIV burden is a global health priority [1, 4–6]. The World Health Organization (WHO) and The Joint United Nations program on HIV/AIDS (UNAIDS) jointly laid out an overarching long-term strategy for overcoming the HIV epidemic entitled ‘Treatment 2.0’ in which the integration of HIV services with non-HIV services is listed as a key goal [7].

Integrated health services provision is gaining increasing importance in the context of declining international assistance for national HIV responses in low and middle-income countries [7]. There is mounting evidence suggesting that vertical HIV service delivery is unconducive to long-term programme sustainability and that the integration of HIV services into general care, reduces services delivery costs as well as duplication and fosters synergies in health systems especially in resource-constrained settings such as Uganda [4–6, 8].

It has been argued that a strong vertical approach was justified during the emergency phase of HIV services scale-up more than a decade ago but that a transition to a more ‘durable approach’ in which HIV programming is integrated in mainstream health systems is crucial [9, 10]. Hence, it is imperative to understand barriers to the integration of HIV services into general out-patient services in order to inform planning for the long-term sustainability of HIV services in Uganda and the broader Sub-Saharan Africa region.

## The HIV and aids epidemic in Uganda

Uganda has a generalized HIV epidemic with an estimated 1.6 million people living with HIV [10]. With the exception of South Africa (23%) and Nigeria (15%), Uganda (10%) has the highest HIV incidence rates in Sub -Saharan Africa [11]. Overall, Uganda had the fourth highest population of PLHIV in SSA in 2013 [11].The National AIDS Indicator survey revealed that national HIV prevalence rates had increased from 6.4% in 2005 to 7.2% in 2011 [12]. In 2015, the Uganda AIDS Commission estimated that the national HIV prevalence rate had risen to 7.9% [12]. In 2015, the number of Ugandans enrolled on ART was 763,720 which represents about 46% of the population in need [12]. Additionally, patients enrolled on ART are living longer, compounding total estimates of future need [13]. It is estimated that the number of people living with HIV in Uganda will rise to 13% by 2020 implying that ART scale-up targets in the country will more than double [13].

Despite the accumulating evidence and policy guidance [4, 6], calling for the integration of HIV services with other health services, vertical HIV clinics are still common in Uganda [14] and the broader Sub-Saharan Africa (SSA) region [15–17]. Several health facilities in Uganda still run specialized HIV clinics across both public and private providers [14].

Under this vertical (stand-alone) model [18], HIV clinics in Uganda run as separate units within health facilities on designated days of the week numbering between 2 and 5 days of the week with a dedicated workforce deployed to these clinics. It is common to find that these HIV clinics have dedicated physical space within health facilities, ranging from permanent physical infrastructure such as patient counselling rooms to semi-permanent structures such as patient shades and tents. Typically, HIV clinics have parallel patient flow and filing systems [19–21]. Whereas vertical HIV clinics are common in Uganda, several health facilities have experimented with health service delivery models in which HIV and non-HIV services are integrated [1].

Although numerous experimental studies have been conducted to assess the implementation experiences of integrating HIV with non-HIV services such as with regard to primary care [22], mental health services [23], maternal and child health services [24], sexual and reproductive health services [25, 26], there is a dearth of evidence exploring *why* vertical HIV clinics persist in SSA despite the accumulating evidence urging the integration of HIV services into mainstream health systems. This paper addresses this gap. We aimed to describe the specific contexts underpinning the endurance of vertical HIV clinics in Uganda.

Moreover, many studies have examined the integration of HIV services from a predominantly health-systems efficiencies lens [25]. The perspectives of patients and front-line service managers are under-explored in current top-down discourses yet the latter perspectives have recently been found to be influential on health services integration outcomes [26].

**Fig. 1:**
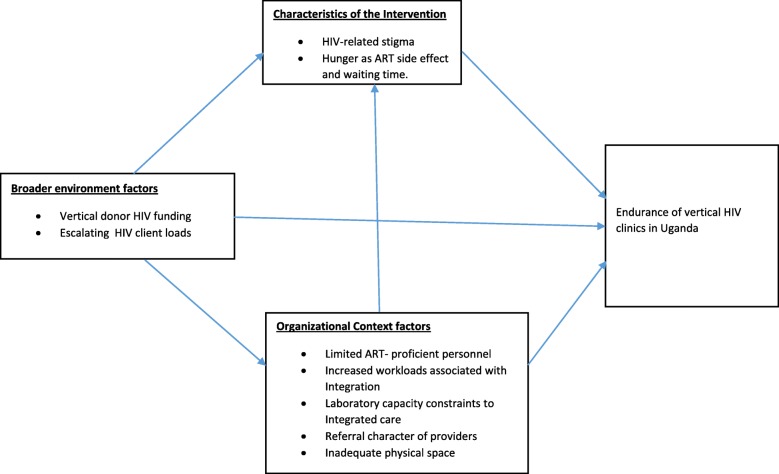
Interactions in factors contributing to vertical HIV clinics. Image adapted from Shediac-Rizkallah & Bone (1998) [27]

This study was guided by the theory by Shediac-Rizkallah & Bone [27] with respect to the three broad themes proposed as influential on health program sustainability; a) characteristics of the intervention b) organizational context factors and c) broader environment factors (Fig. 1).

## Methods

### Research design

This was a descriptive qualitative study conducted between April and June 2016. We adopted a qualitative approach aimed at gaining an in-depth understanding of the specific contexts underpinning the endurance of vertical (stand-alone) HIV clinics in the health system in Uganda from the perspective of service providers and patients.

### Study sites and selection

Health facilities were selected in a two-stage process. In stage one, a nationally-representative sample of 195 (out of 394) health facilities which participated in Uganda’s emergency national anti-retroviral therapy (ART) roll-out phase (2004–2009) [28, 29] were selected from the published Ministry of Health Report listing accredited ART sites as at March 2010. The detailed sample selection procedure for this study phase is described elsewhere [14].

In Stage Two, from the 195 health facilities selected from stage one [14], we purposefully selected 16 health facilities which run vertical or specialized HIV clinics. A vertical HIV clinic was defined as having a stand-alone HIV clinic running on designated days of the week, with a dedicated workforce, and having separate physical space from the rest of the health facility. We selected at least 2 health facilities from each of Uganda’s 8 geographic sub-regions (South West, East Central, Mid-East, Mid-West, Central 2, Mid North, North East and Kampala) as defined by The Uganda Bureau of Statistics [30]. As shown in Table 1, we aimed to achieve diversity in our sample with regard to ownership-type (public /private), setting (rural /urban) and level of care (tertiary/ secondary) in the Ugandan health system [31].

**Table 1:** Characteristics of participating health facilities

	ACCRONYM	OWNERSHIP	LEVEL OF CARE [31]	SETTING	SUB-REGION	ANNUAL ART PATIENT VOLUMES (As at June 2015)
1	PUB-001	PUBLIC	Referral Hospital	Urban	South West	24,408
2	PUB-002	PUBLIC	Referral Hospital	Urban	Kampala	2408
3	PUB-003	PUBLIC	Referral Hospital	Urban	Central 2	6414
4	PUB-004	PUBLIC	District Hospital	Urban	East Central	598
5	PUB-005	PUBLIC	Health centre IV	Peri-urban	Mid-East	458
6	PUB-006	PUBLIC	Health centre IV	Rural	Mid-north	2034
7	PUB-007	PUBLIC	Health centre IV	Rural	East-Central	263
8	PUB-008	PUBLIC	Health centre IV	Peri-urban	Mid-west	298
9	PUB-009	PUBLIC	Health centre IV	Rural	North East	126
10	PNFP-001	NOT FOR PROFIT	Referral Hospital	Urban	Kampala	4337
11	PNFP-002	NOT FOR PROFIT	Referral Hospital	Urban	East central	1727
12	PNFP-003	NOT FOR PROFIT	Health Centre IV	Rural	Mid-East	647
13	PNFP-004	NOT FOR PROFIT	Health Centre IV	Peri-urban	South West	402
14	PFP-001	FOR-PROFIT	Health centre III	Urban	Mid-West	324
15	PFP-002	FOR-PROFIT	Health Centre II	Urban	Central 2	29
16	PFP-003	FOR-PROFIT	Health Centre II	Rural	Mid-North	46

### Data collection

#### Structured questionnaire

The demographic characteristics of participating health facilities (Table 1) were constructed based on data generated from a structured questionnaire which was self-administered by the head of the HIV clinic at each of the 16 health facilities. This paper is derived from a broader mixed-methods study of the sustainability of ART scale-up implementation in health facilities in Uganda [14, 32, 33]. Details of the structured questionnaire are described elsewhere [14, 29, 32, 33].

#### Semi-structured interviews

A semi-structured interview guide was constructed based on the three groups of factors suggested as potentially influential on health program sustainability [27] namely; a) characteristics of the intervention b) organizational context factors and c) broader environment factors.

A total of 78 semi-structured interviews (SSIs) were conducted with the head of HIV clinic in each of the 16 health facilities, two of the most experienced clinicians at each of the participating health facilities (*n* = 32) as well as the facility in-charge (*n* = 16). The face-to-face Interviews were conducted in English in the offices of participants on-site. In addition, a data validation workshop [34] (Table 2) was conducted followed by individual interviews with the head of the HIV clinic of 14 of the participating health facilities. Interviews were conducted by the first author and four research assistants experienced in qualitative interviews with a background in health services research.

**Table 2:** Background characteristics of participants

Health worker respondents	*n = 78*
Head of ART clinic (16), facility in-charges (16)	*n = 32*
Clinicians	*n = 46*
Sex	
Male	41
Female	37
Age (years)	Range: 22–59
	Median 36
Work experience	Range: 1–20
	Median: 5.4
Focus Groups (ART Clients)	*n = 68*
Sex	
Male	37
Female	31
Age (years)	Range 38–59
	Median: 44

#### Focus group discussions

Eight focus group discussions (FGDs) were conducted with a total of 64 clients selected from HIV clinics in our study sample. At least one patient FGD was conducted in each of the eight geographic sub-regions of Uganda [30].

#### Selection of participants

Within the health facilities, a convenience sample of clients attending the ART clinic for scheduled routine reviews was selected. Participants were selected from those who had completed their review sessions. Within the patient waiting area, the ART clinic in-charge introduced the investigators, who provided an overview of the study to clients. Subsequently, clients were invited to participate in the study on a voluntary basis. We enrolled 6–8 clients who expressed willingness to participate in the study ensuring an appropriate mix of experience on the ART program (those enrolled for several years and those with a shorter enrollment history). We sought to achieve gender-balance in the composition of all the FGD groups. Hence, the FGD groups involved a mix of genders. The focus group discussions were conducted using topic guides framed around Shediac-Rizkallah’s framework [27]. The FGDs were conducted in a private room within the health facilities by the first author and two note takers and lasted about one and a half hours on average.

### Data analysis

Interviews and focus group discussions were audio-recorded and transcribed verbatim by a professional transcriber prior to thematic coding.

The thematic analysis was conducted in a two-stage process involving both inductive and deductive approaches. In the first stage, a code book was generated inductively following multiple readings of interview data by the first author for data familiarization [35]. The code book was then reviewed by three authors (JR, JK1, JK2) [35, 36]. After incorporating in-put from three authors, the resulting codebook was then applied to all interview data. In the second stage, the emergent codes were categorized under the three broad themes proposed by Shediac-Rizkallah & Bone [27] (*Intervention characteristics, organizational context, broader environment factors*) as influential on health program sustainability. The final analyses were arrived at through a consensus process involving all the authors. Additionally, this study implemented the procedures [34] recommended for ensuring rigour in qualitative research as shown in Table 3.

**Table 3:** Processes for ensuring rigour in case-study analysis adapted from Gilson et al. (2012) [30]

PRINCIPLE	
Prolonged engagement	Multiple on-site visits were made to the case-study facilities. Investigators engaged in informal discussions with clinicians and HIV clinic managers as well as conducting formal, face-to-face interviews with multiple informants per health facility.
Use of theory	This study draws upon the analytical framework by Shediac-Rizkallah & Bone (1998).
Case selection	Sixteen health facilities which run a stand-alone HIV clinic were purposefully selected from a nationally-representative sample of 195 health facilities across Uganda participated in the pilot national ART roll-out phase.
Sampling	We aimed to have a sample that had appropriate representation of health facility demographics in Uganda with respect to a) setting(rural/urban), b) ownership-type(public, for-profit, not-for-profit) c) Level of care(tertiary, secondary, primary).
Multiple methods	Multiple methods were used including face-to-face interviews, a structured questionnaire and informal engagements with clinicians and the head of the ART Clinic
Triangulation	Case descriptions were constructed based on triangulation across multiple data sources (Questionnaire data and, interviewee data).
Peer debriefing and support	Data analysis involved a team-based process involving at least three authors.
Respondent validation	A data validation workshop was conducted with involving the head of the HIV clinic in 14 of the participating health facilities.

## Results

The results emerging from this study are presented under the three broad themes suggested by Shediac-Rizkallah & Bone [27].

### Characteristics of the intervention

#### HIV-related stigma

HIV-related stigma among healthcare providers was consistently cited as the motivation behind the establishment of specialized HIV clinics based on the HIV services implementation experiences of participating health facilities. Six health facilities (PNFP-001, PNFP-002,PUB-004, PUB-007,PUB-008, PUB-009), indicated that they initially implemented service delivery models in which all health services were integrated prior to the phase of national ART roll-out between 2004 and 2008 [28]. They reported that during this period, when HIV and non-HIV services were integrated, HIV patients frequently suffered provider stigma and were discriminated against by health workers who were reluctant to handle them due to a perceived risk of exposure to HIV infection and that they were frequently segregated and served last. As a result, the HIV serostatus of clients was often inadvertently breached. Additionally, patients reported enduring longer waiting time compared to non-HIV patients.

*‘At first when we started HIV services we could prescribe drugs and send our patients to the general hospital pharmacy to get drugs but then there was a problem of stigma. Also, the waiting time was long because most patients at this hospital pay for the services but HIV services were mostly free so they would work on the paying patients first and then handle HIV patients last which stigmatized clients ‘[Interviewee 2, PNFP-001].*

*‘People with HIV suffer discrimination. There is a lady health worker in the labour ward who does not attend to HIV positive mothers. She promptly refers them to the district hospital because she says they will infect her. She cannot touch their blood if she is on duty’ [Patient 121, PUB-008].*

Overall, HIV clinic managers perceived patients seeking care in specialized HIV clinics as suffering less stigmatization compared to integrated care models due to a shared seropositivity status among clients attending HIV clinics, peer-support group dynamics as well as a dedicated workforce in HIV clinics who were frequently trained and had come to terms with serving HIV patients without fear of infection. In the focus group discussions, patients indicated that they had, over time, developed rapport with HIV clinicians who had gained an intimate knowledge of their individual cases which would be difficult to sustain in a general outpatient department.

*‘HIV infected clients attending HIV clinics require a ‘one-on-one touch’ that may be difficult to offer in general clinics’ [Interviewee 3, PUB-006].*

##### The need for patient privacy in HIV services

Related to the theme of stigma, both clients and providers raised the need for patient privacy and confidentiality during routine counselling sessions on ART clinic days and justified the need for constructing specialized counseling rooms within HIV clinics for this purpose. They emphasized that this was necessary to ensure patient openness during counseling sessions which ensured that clinicians made accurate choices of care. Antiretroviral therapy (ART) was described as a complex intervention requiring many facets of care. The need for counseling was consistently raised as a distinguishing feature between HIV and non-HIV services. This necessitated specialized rooms for counselling patients as part of routine care and was frequently cited as a justification for erecting enclosed rooms to ensure patient confidentiality and to foster client candor during routine counselling sessions.

*‘General clinics do not have dedicated counsellors who are critical to the care processes for HIV-infected clients right from diagnosis, through to start of treatment, adherence patterns and follow up with adverse drug reactions among others’ [Interviewee 1,PFP-002].*

HIV clinic managers described vertical clinics as having emerged organically from the evolution of HIV service delivery over the last 15 years rather than as an outcome of long-term planning. Participants from a not for-profit hospital (PNFP-001) described the origins of their HIV clinic as emanating initially from a push for a specialized pharmacy for HIV patients.

*‘We then advocated for having our own store for ARVs (antiretroviral drugs) which was later expanded into the clinic. When we opened up the place (HIV clinic) people flocked in because they liked the privacy because we were located in a hidden part of the hospital’ [Interviewee 2, PNFP-001].*

##### Hunger and waiting time

In the focus group discussions with patients, hunger was highlighted as one of the attributes uniquely affecting HIV patients due to the side effects of their medication and one of the factors justifying specialized HIV services. Patients indicated that they suffer hunger more frequently than non-HIV patients due to the nature of their medication which they described as appetite-inducing and that they were less able to tolerate long waiting times. A regional referral hospital participating in the study (PUB-002) reverted back to an integrated services delivery model after the withdrawal of a major donor grant for ART services. Patients recalled that when HIV services were integrated into general outpatient (OPD) services, the waiting times for HIV services became longer compared to the time when the hospital operated a stand-alone HIV clinic.

*‘That time we had challenges with getting laboratory services in the OPD (out patients department) because the place was highly congested and we were there with other patients so we had to follow one queue and they were not considerate of us since we face severe hunger after waiting for long hours unlike other patients’[Patient 126, PUB-002].*

### Organizational context

#### Health-system barriers to service integration

##### Increased workloads associated with service integration

A consistent theme across our interviews with HIV clinic managers were concerns over the rapidly increased health worker workloads associated with implementing integrated service delivery models. Representatives from a section of health facilities (PUB-002, PUB-007, PUB-008, PUB-009) reported being overwhelmed by the surge in workload during the course of implementing integrated care. They described the expectations on health workers as unrealistic as the implication was that they were suddenly required to be proficient at treating a wide range of ailments.

*‘The guidelines are changing so fast but the health system is remaining more less the same. If you see what is required of a health worker to handle a single HIV patient. The workload is dizzying. Do you have a cough today? Then I am supposed to fill the presumptive TB register. Then fill a laboratory request form. Conduct counselling, clinical examination, cancer screening. In addition, I have to go back and fill the HIV forms’ [Interviewee 1, PUB-005].*

*‘The workload on each individual health worker increases when you implement integrated services because it would mean that health facilities provide all services; malaria, nutrition, family planning together. It means you are the doctor to review the patient, you are the TB specialist, you are the eMTCT specialist, you take off my blood for CD4 and viral load. That while you are seated in that chair, you can handle all of my needs so that you avoid the client walking at different points in the clinic. Can we cope with such a workload? [Interviewee 1, PUB-007].*

Health workers in HIV clinics (PUB-006, PUB-007, PUB-008, PUB-009) implementing integrated health information systems across disease profiles revealed that the resulting workload was burdensome. They described the tasks of having to fill multiple manual medical forms as required by Uganda’s ministry of health, in the bid to unify the disparate records across multiple disease interventions and thematic areas such as Tuberculosis (TB), Prevention of Mother to Child Transmission of HIV (PMTC) and cancers into a merged manual data record system, as laborius.

##### Shortage of ART-proficient health workers

The shortage of ART-proficient personnel in participating health facilities was identified as a barrier to HIV services integration into general care. HIV clinic managers described the clinical management of HIV as requiring specialized knowledge and expertise. This was said to be critical in ensuring that a high quality and a good standard of HIV care is offered. They mentioned task-shifting to mid-cadre such as nurses and lower cadre such as lay workers as having been facilitated by periodic refresher trainings in HIV management and that designating all health workers in the health system overnight as HIV clinicians would be infeasible.

*‘Human resource challenges remain critical. HIV work is specialized and labor intensive in view of the multiple complexities that clients have. Integrating HIV services with non-HIV services will call for additional reinforcements in human resources to manage the demand. Additionally, there is need for continuous medical training for all those involved in HIV care to ensure that they keep abreast with the rapidly changing treatment and care algorithms in order to offer quality services’ [Interviewee 2, PNFP-002].*

In addition, participants pointed out that the sheer volume of HIV patients is burdensome on its own and that combining it with the general pool in the out-patient department (OPD) as a development that would overwhelm the regular workforce.

*‘Most HIV clinics from the sub-district level and above have huge client loads for HIV clients and yet the human resource capacity has not been supported in commensurate ways. It is very difficult to integrate the general HIV clinic in say the entire OPD and have them attended to in a pool of general OPD clients with the limited human resource capacity’[Interview 3, PUB-004].*

##### Laboratory capacity barriers to service integration

HIV clinic managers frequently mentioned the specialized laboratory and diagnostic needs of HIV care and treatment as a justification for vertical HIV service delivery. Sharing of laboratory resources between HIV and non-HIV services was described as problematic due to the relatively lengthy processes involved in running HIV tests such as CD4 count tests as compared to say, tests for malaria or pregnancy screening. HIV clinic managers and patients perceived specialized HIV tests as taking longer than the average time taken in non-HIV tests.

*‘The problem with the laboratory is that we share it with the OPD (Out Patients department). We have to run blood smear and malaria parasites tests and at the same time do CD4 tests. The CD4 machine takes 30 minutes to run tests for one person so if you have 30 people it will take the whole day’[Interviewee 1,PUB-008].*

In the 16 participating health facilities, six providers (PNFP-001, PNFP-002,PUB-004, PUB-007,PUB-008, PUB-009) recounted experiences of experimenting with HIV services integration into general care after the end or withdrawal of major HIV service delivery donor grants and the challenges experienced with transiting from vertical HIV laboratory services to a fully integrated hospital laboratory services model. HIV clients in a Regional Referral Hospital (PUB-002) undergoing these changes were especially unequivocal.

*‘They would often draw blood from you but the results would never come out as fast as they did when we had the ART Clinic. In fact, after our donor withdrew, it was impossible to get CD4 count results from the general hospital laboratory’ [Patient 128, PUB-002].*

*‘There was a moment of confusion. Patient blood samples for CD4 tests were frequently lost in the general hospital laboratory. They would draw your blood from the main hospital laboratory under a request from a health worker in the ART clinic. The requesting ART clinic staff would not be around to follow up on samples. Even when you yourself followed up with the main Laboratory there was no one to ask since they did not originate the laboratory request. It was a moment of chaos. [Patient 121, PUB-008].*

##### Referral character of tertiary hospitals

Five of the 16 health facilities running vertical HIV clinics were tertiary hospitals in the Ugandan health system which operates on a referral basis [31]. The representatives of tertiary hospitals (PUB-001, PUB-002, PUB-003, PNFP-001, PNFP-002) justified the continued operation of specialized HIV clinics by the referral character of their hospitals. Patients requiring more advanced clinical care from lower-level health centres were frequently referred to tertiary hospitals from lower-tier health facilities especially patients not achieving viral suppression.

*‘Being the regional referral hospital, we continually get patients referred to us from lower-level health centres especially those who have failed on first line and second line drugs and need more advanced clinic care which lower health centres aren’t able to handle. We are a regional centre of excellence and have expertise to offer advanced HIV care’ [Interviewee 1, PUB-001].*

Within our sample of 16 health facilities (Table 1), tertiary hospitals more than any other category of health facilities reported the need to run vertical HIV clinics as a specialized unit to which other units within the hospital referred patients needing expert HIV care and treatment. This included HIV-associated ailments such as Tuberculosis (TB) which were housed within the HIV clinic.

*‘We have the TB clinic here within our HIV clinic complex. Our TB clinic is a one-stop shop. All TB cases in the hospital come to us. When we test you and find you are HIV positive, you remain with us. If you are HIV positive we follow you up’ [Interviewee 1, PNFP-001].*

Providers justified the need for specialized HIV care at the tertiary level on the need for ‘centres of excellence’ in HIV care and HIV research sites that cannot be hosted at primary or even secondary level of care in the Ugandan health system [31].

*‘Specialized HIV clinics are critical especially in referral settings. As people stay on treatment for several years, there are several adherence, toxicity, drug failure issues that are coming up on a day-to-day basis. These need to be managed critically to avoid future treatment failure challenges with the limited treatment options’ [Interviewee 3, PNFP-002].*

##### Inadequate physical space

A constraint which was frequently cited by HIV clinic managers was the inadequacy of physical space to implement integrated care models given the physical infrastructure challenges reported by the majority of participating facilities. Dedicated HIV clinics were reported as a strategy of responding to physical space limitations. A section of health facilities, especially lower-level ones, reported that the ART clinics were designated on specific days of the week as a deliberate strategy. The rationale behind designating ART clinic days was partly because this is when substantial physical space in health facilities could be devoted to accommodating the typically long patient queues which couldn’t be sustained more frequently during the week. Because of a specialized patient flow system at HIV clinics and escalating caseloads, patients often have to wait in long queues which necessities the construction of waiting tents at ART clinics. All these factors were said to impose new demands on the physical infrastructure of health facilities which posed a barrier to implementing integrated service delivery models.

*“The space is not enough. We have one tent for the patients and even some of the client counselling is done in the open which makes the patients uncomfortable.” [Interviewee 3, PUB-007].*

At a mission hospital (PNFP-001), clinicians complained of sharing counselling rooms whereby two patients are concurrently counseled by two separate counsellors in small rooms and as such clients could hear each other’s counseling sessions. Clinicians indicated that this compromised client privacy and this impeded patient candour during on-going counselling sessions.

### Broader-environment factors

#### HIV funding architecture

Interviews with HIV clinic managers illuminated the influence of external donor funding architectures on the continued verticalized HIV programming in Uganda. The President’s Emergency Fund for AIDS Relief (PEPFAR)‘s funding model in Uganda was consistently cited as an example of international assistance frameworks that perpetuate a vertical HIV approach owing to U.S. government global health diplomacy priorities in Uganda. In a country that spends less than 3% of its health sector budget on the national HIV response [37], PEPFAR was reported to have sustained substantial HIV-specific funding support in Uganda for more than a decade.

*‘There is a historical aspect to this. HIV services in most of the health facilities in Uganda were introduced in a vertical fashion with heavy donor funding. There has also been a heightened focus on HIV treatment roll out in an effort to test and treat all HIV infected persons which has promoted the growth of vertical clinics’ [Interview 1, PFP-003].*

PEPFAR was said to be providing wide-ranging HIV-specific support including mass recruitment of health workers specifically for HIV service delivery, on-site support supervision for HIV services, laboratory sector support principally aimed at HIV services and periodic trainings of health workers in ART standard-of-care. This HIV support was said to be vertically channeled through ‘implementing partners’ and not directly into the health system as a whole coupled with parallel reporting structures. However, vertical HIV funding was not only reported from bilateral and multilateral sources but increasingly from private individuals and foundations. The availability of philanthropic aid for HIV services at the facility-level was also highlighted as one of the factors fostering stand-alone HIV clinics.

*‘One of the reasons why we have been able to sustain HIV treatment for all this time is our funders from the United Kingdom, from Canada, from United States. For instance, they have been funding treatment of opportunistic infections (OIs) and other care costs which are not catered for by our main funders’ [Interviewee 1,PNFP-001].*

*‘Individual donors from the West made it possible to re-model a former laundry room for hospital staff into the present HIV clinic building. The central hospital management was inspired to start dedicated HIV services with donor support’ [Interviewee 3, PUB-002].*

#### HIV demand-side factors

The growing case load of HIV patients in Uganda since June 2004 coupled with the frequent lowering of ART enrollment thresholds were jointly raised by HIV clinic managers as ‘demand-side’ factors that necessitated dedicated HIV services. Participants argued that the continually-increasing demand for HIV services couldn’t be adequately met in general OPD (out-patient) departments. A consistent theme in the interviews with clinic managers was the notion that vertical HIV clinics were unplanned but arose out of escalating patient volumes over time. The following quotes illustrate this:

*‘There was no HIV clinic to start with. Services began as a three-hour service, once a week on a Monday. There was no ART at the time. Essentially it was management of opportunistic infections (OIs) and counselling. The three-hour services were provided in a large room which was partitioned into two to accommodate clinicians and counsellors. The key driver for transforming into a fully- fledged clinic was the consistent increase in patient numbers’ [Interviewee 2, PNFP-001].*

*‘At the beginning it was a one day clinic. When the patient numbers grew bigger, we improvised one of the rooms under an un-used theatre. We partitioned the room into two. One for the counsellor and another for the second counsellor. We started with 10 clients then the number rose to 69 and subsequently to 300 patients. In terms of staffing, the clinic began with 3 staff which rose to 10 and then 50. Staff were seconded to the HIV clinic when the demand for HIV services increased’ [Interviewee 1, PUB-004].*

In a not for-profit hospital (PNFP-001), patients rose from 69 in 1988, to 2400 in 2010 and to almost 5000 in June 2015. The dramatic increase in patient volumes in participating health facilities since initial ART roll-out in 2004 and the substantial HIV client loads across our sample (Table 1) highlights the role of sustained demand for dedicated HIV clinics in Uganda.

## Discussion

There have been numerous studies exploring the implementation experiences of integrating HIV services into mainstream health systems [4–6]. We aimed to describe the specific contexts underpinning the persistence of stand-alone HIV clinics in Uganda despite mounting calls for the integrated delivery of health services. Our study demonstrates that vertical HIV clinics have endured in Uganda in response to concerns of provider stigma under integrated service delivery models, the specialized needs of HIV disease management [38] that necessitate a dedicated workforce, health-system capacity constraints associated with implementing integrated service delivery models such as the shortage of ART-proficient personnel and laboratory capacity constraints, escalating HIV patient volumes and external funding architectures that perpetuate verticalized HIV programming. Participants described stand-alone HIV clinics as having emerged organically from the evolution of HIV service delivery over the past decade in Uganda rather than as outcome of long-term planning.

Although there is a widespread notion in the literature suggesting that verticalized HIV programming represents inefficient resource allocation and constitutes a missed opportunity for promoting synergies in health systems as a whole, our findings elicit a more nuanced picture. From the perspective of providers, HIV clinics were described as a pragmatic strategy for optimizing the limited ART-proficient workforce, managing the escalating demand for HIV services and responding to systemic challenges of an inadequate service delivery infrastructure characterized by a shortage of physical space and a weak laboratory capacity. Our findings suggest that in countries with a dual challenge of generalized HIV epidemics and under-resourced health systems, such as Uganda, a vertical approach may continue to be necessary. In a recent systematic review, Topp and colleagues [1], conclude that low-income countries are unprepared for implementing integrated care models and propose five health-systems thematic prerequisites that need to be in place before health services integration can become a reality. These include putting in place; (1) the organizational framework of frontline services (2) health worker preparedness (3) community and client preparedness (4) upstream logistics and supply (5) policy and governance issues.

Our data support the perspective by Atun and colleagues [9] that there is a place for vertical HIV clinics in health systems and highlights the complex nature of barriers standing in the way of the global health goal of integrating HIV with other health services and the need for more nuanced approaches as opposed to what Church and colleagues [16] have termed ‘blanket integration policies’. In this connection, Smit and colleagues [5] have called for a more ‘incremental approach’ in achieving the integrated service provision agenda.

### The debate on HIV-related stigma

There is an ongoing debate in the literature regarding whether patients suffer less HIV-related stigma in vertical HIV clinics as compared to under care models where health services are integrated [4, 15]. Contrary to previous studies which suggest that stigma is higher in stand-alone HIV clinics [39, 40], in our sample of health facilities, both patients and providers concurred that clients attending their specialized HIV clinics experienced less provider stigma compared to the period when these health facilities implemented integrated care. In this regard, our study agrees with a study by Church and colleagues [16] in Swaziland which compared HIV-related stigma in vertical and under integrated services models and found that the notion that stigma is higher in vertical HIV clinics did not hold. Similar findings have been reported by Colombini and colleagues [41]. However, our findings suggest that provider HIV stigma is widespread in the Ugandan health system and that policy responses in this regard are warranted.

Previous studies have reported the integration of HIV and non-HIV services under experimental settings [4, 15–17]. This study documents provider experiences of service integration under naturalistic or non-experimental settings. An important contribution of this study is that we report dual experiences by providers of implementing both integrated service delivery models and stand-alone HIV services in non-experimental settings [42] thereby eliciting a unique comparative lens from both a provider and patient perspective .

In this study, participants justified stand-alone HIV clinics as necessitated by the specialized nature and the ‘complexity of HIV disease management’ [15, 38]. There have been similar characterizations in the literature of ART being a ‘complex intervention’ [43, 44]. Previous studies have found that HIV patients are uniquely affected by hunger due to the nature of their medication and yet long waiting times are typical in HIV care in high-prevalence countries [44–46]. Our finding that patients reported longer waiting times after HIV services were integrated into OPD services agrees with a study from Kenya [15]. There are studies urging caution in the integration of HIV services in general care over concerns that this could potentially undermine the effectiveness of HIV programmers [21, 25, 47].

### The influence of donor funding architectures

Our findings demonstrate that the current mode of HIV funding in Uganda is highly donor-dependent. Over the past decade, the majority of external donor support has been provided under HIV-specific assistance frameworks as opposed to a sector-wide approach. Participants identified donor support that is solely focused on HIV activities or vertical funding as a factor that directly sustains stand-alone HIV clinics in the Ugandan health system.

On the other hand, it has been argued that vertical HIV programming is rooted in ‘AIDS exceptionalism’ or a higher priority accorded to HIV causes in comparison to other disease responses [48, 49]. As some scholars have argued, the enduring verticalized HIV programming is sustained by external HIV financing especially from Global Health Initiatives (GHIs) [14, 50–52, 53].

### Health-system capacity constraints

Our study illuminates the deficiencies in capacity of an under-resourced health-system to implement integrated health care (Fig. 1). The bottlenecks identified in this study include the shortage of ART-proficient personnel and laboratory sector capacity constraints in concurrently managing HIV and non-HIV services needs due to the peculiarities of the former. This study adds to the sparse but growing literature eliciting these barriers [1, 10], including the workforce constraints associated with integrated care provision [6, 10]. To promote service integration, health-system strengthening interventions are critical. These include strengthening laboratory capacity at the facility-level or through supporting laboratory ‘hubs’ at sub-national level to enhance the capacity of providers at lower-level health facilities to simultaneously conduct HIV and non-HIV investigations. Training programs in HIV management across the breadth of the health workforce as opposed to focusing on dedicated personnel in HIV clinics could potentially promote capacity for HIV services integration into general care especially at the primary care level. Smit and colleagues [2] have called for training programmes in implementing integrated systems of care targeted at health workers and facility in-charges to increase uptake.

### Differentiated HIV care models and prospects for integrated care

In this study, we found that health facilities were overwhelmed by the demand for HIV services and that this was identified as a barrier to integration owing to the sheer volume of HIV patient loads that necessitated dedicated personnel and resources. This study adds to mounting calls for the scale-up of differentiated care models (DCMs) [54] in HIV care which are partly aimed at decongesting HIV clinics through spacing appointments for stable HIV patients and reducing clinic-based care in favour of more community-based care platforms [33, 55, 56]. DCMs allow for optimization of the limited clinician cadre in Sub Saharan Africa by permitting ‘stable’ patients to be handled by non-clinician personnel including co-opting community health workers in ART management, as well as expert patients, under community-supported models of care [56]. The roll-out of differentiated care models across Sub-Saharan Africa could reduce pressure on over-burdened health systems, reduce the costs of service delivery [54] and promote service integration through realizing dramatic reductions in HIV outpatient burdens that would in turn allow more attention and resources to non-HIV services. Our study concurs with calls by Duncombe and colleagues [55] of resolving health system implementation barriers to DCMs roll-out in countries with generalized HIV epidemics. In the context of reports of declining international assistance for HIV services scale-up and mounting questions on long-term program sustainability [32, 33], calls for differentiated care scale-up are especially timely.

### Limitations

We purposively selected 16 health facilities which run specialized HIV clinics from a national sample of 195 health facilities in Uganda. Hence, we did not aim for statistical generalization of our study findings. Rather, we aimed for in-depth, insights into the contexts underpinning the persistence of HIV clinics in the Ugandan health system to gain a facility-based understanding of this phenomenon. Additionally, the selected health facilities were drawn from those which participated in the pilot phase of national ART roll-out in Uganda. ART roll-out in Uganda commenced at a relatively higher level of care in the Ugandan health system between 2004 and 2009. Although our case-study sample was broadly representative of HIV service delivery characteristics, especially with respect to level of care, during the latter period, the 16 selected health facilities may not be fully representative of current HIV service provision in Uganda. This study however had many strengths. Our study contributes to filling the void in the literature attempting to understand *why* stand-alone HIV clinics endure in the Ugandan health system. Relative to the current literature, we report comparative experiences by providers of implementing both vertical HIV clinics and integrated care. Although much of the current evidence base on integrated systems of care is drawn from experimental research, the unique contribution of this study is in documenting naturalistic experiences by providers and patients of the dual implementation experiences of vertical HIV clinics and integrated service provision under non-experimental conditions.

## Conclusion

Our study offers in-depth, contextualized insights into the factors contributing to the endurance of vertical HIV clinics in Uganda. Our analysis suggests that there is a complex interaction in supply-side constraints (shortage of ART-proficient personnel, increased workloads, laboratory capacity deficiencies) and demand-side factors (escalating demand for HIV services, psychosocial barriers to HIV care) as well as the specialized nature of HIV disease management which pose challenges to the integrated services agenda.

## Abbreviations

AIDS: Acquired Immune Deficiency Syndrome
ART: Anti-retroviral therapy
ARVs: Anti-retroviral
GHIs: Global Health Initiatives
HIV: Human Immunodeficiency Virus
PEPFAR: The Presidents’ Emergency Fund for AIDS Relief
SSA: Sub-Saharan Africa
UNAIDS: The Joint United Nations Program on HIV/AIDS
WHO: World Health Organization
